# EZH2 Inhibition and Cisplatin as a Combination Anticancer Therapy: An Overview of Preclinical Studies

**DOI:** 10.3390/cancers14194761

**Published:** 2022-09-29

**Authors:** Ivana Samaržija, Marko Tomljanović, Renata Novak Kujundžić, Koraljka Gall Trošelj

**Affiliations:** Laboratory for Epigenomics, Division of Molecular Medicine, Ruđer Bošković Institute, 10000 Zagreb, Croatia

**Keywords:** cisplatin, EZH2, EZH2 inhibitors, drug resistance, combination therapy, targeted anticancer therapy, lung cancer, ovarian cancer, breast cancer, testicular germ cell tumors

## Abstract

**Simple Summary:**

Cisplatin is a chemotherapy drug widely used in the treatment of different cancer types. However, cisplatin displays a high range of toxicity and its use usually leads to resistance. Therefore, combination therapies that include cisplatin are explored to alleviate the problems that are elicited by cisplatin use. EZH2 is an epigenetic regulator with an increased expression and activity in many cancer types, which, in general, potentiates cancer growth and expansion. In the past decade, many EZH2 inhibitors were introduced and investigated for their anticancer properties. In this review paper, we explore the work that analyzed the joint action of EZH2 inhibitors and cisplatin in different tumor types. We found that combination therapy of EZH2 inhibitors and cisplatin could potentially be beneficial for the treatment of lung, ovarian, and breast cancers. However, in testicular germ cell tumors, according to the published data, such a combination could potentially have antagonistic effects.

**Abstract:**

Anticancer monotherapies are often insufficient in eradicating cancer cells because cancers are driven by changes in numerous genes and pathways. Combination anticancer therapies which aim to target several cancer traits at once represent a substantial improvement in anticancer treatment. Cisplatin is a conventional chemotherapy agent widely used in the treatment of different cancer types. However, the shortcomings of cisplatin use include its toxicity and development of resistance. Therefore, from early on, combination therapies that include cisplatin were considered and used in a variety of cancers. EZH2, an epigenetic regulator, is frequently upregulated in cancers which, in general, potentiates cancer cell malignant behavior. In the past decade, numerous EZH2 inhibitors have been explored for their anticancer properties. In this overview, we present the studies that discuss the joint action of cisplatin and EZH2 inhibitors. According to the data presented, the use of cisplatin and EZH2 inhibitors may be beneficial in the treatment of lung, ovarian, and breast cancers, since there is a substantial amount of published evidence that suggests their concerted action. However, in testicular germ cell tumors, such a combination would not be recommended because cisplatin resistance seems to be associated with decreased expression of EZH2 in this tumor type.

## 1. Introduction

Numerous mutated genes and altered pathways offer cancer cells an arsenal of options for sustaining their growth and malignant potential, including developing resistance to applied therapy [1]. Therefore, very often anticancer monotherapy is insufficient to eliminate cancer cells completely. Combination therapies have been explored since 1951 [2], in an effort aimed at influencing several cancer traits at once [3,4]. Combination therapy is particularly beneficial when cancers become resistant to a given monotherapy, which is an often-seen scenario. In the post-genomic era, combination anticancer therapy is a step toward personalized medicine. Its application represents an improvement compared with a conventional chemotherapy approach where all patients are given the same drug, regardless of the mutational background of the tumor [5,6,7]. Another benefit of combination therapy is that it reduces the cost and accelerates the development of new anticancer strategies, since it is based on the novel roles of existing and approved therapeutics [4]. For all these reasons, combination therapies have been a focus of intensive research in recent years [8].

Combination drug therapies include chemotherapy, targeted cancer therapy, and, more recently, immunotherapy. Depending on the cancer type, different combinations have entered clinical practice and many more are being studied in preclinical models. In this work, we provide an overview of the preclinical studies based on the combined application of cisplatin (a conventional chemotherapy agent) and EZH2 (Enhancer of Zeste Homolog 2) inhibitors (targeted epigenetics therapy) in different cancer types. We discuss published data documenting their synergistic or additive modes of action. We also list scenarios in which this approach induces antagonistic effects. The aim of this review is to present the literature data that may be relevant for future cancer treatment approaches combining the use of cisplatin and various EZH2 inhibitors.

## 2. Cisplatin in Anticancer Therapies

Cisplatin, cisplatinum, or cis-diamminedichloroplatinum (II) is a platinum compound first synthesized in 1845. Its chemical structure was elucidated in 1893. However, only by the end of the 1960s, when it was noted that it had cell division inhibiting properties, did cisplatin attract the attention of cancer researchers. Subsequently, cisplatin became the first FDA-approved (1978) platinum compound for cancer treatment. In the 1980s, it was an example of a successful anticancer drug. Cisplatin has been used for the treatment of many human cancers, including lung, cervix uteri, lymphoma, leukemia, sarcoma, bladder, head and neck, breast, ovarian, and testicular cancers [9,10]. It is still used as the first-line chemotherapy for treating some of these cancers [10].

Cisplatin is a cytotoxic drug which kills cancer cells by damaging DNA by generating DNA-platinum adducts and activating the DNA damage response simultaneously with inducing oxidative stress, p53 signaling, and cell cycle arrest. Cisplatin inhibits DNA synthesis and mitosis, and induces mitochondria-mediated apoptosis. Its effects on dividing cells are non-selective and are associated with severe adverse effects. Therefore, cisplatin displays relatively high levels of systemic toxicity. Indeed, cisplatin induces hepatotoxic, nephrotoxic, cardiotoxic, neurotoxic and/or hematotoxic damage, allergic reactions, increased susceptibility to infections, gastrointestinal disorders, hemorrhage, and hearing loss [9]. Besides its high toxicity mediated by the formation of the DNA-platinum adducts and several other mechanisms that include nuclear and mitochondrial DNA, and also inflammatory responses and other survival pathways, another obstacle to cisplatin use is the frequent development of resistance, which calls for additional therapeutic intervention.

### Resistance to Cisplatin

Resistance to cisplatin is a relatively widely studied and well-explored research field, with many of the resistance-mediating mechanisms having been elucidated. Since these mechanisms are potentially important for understanding the results of combined therapies that include cisplatin, they are presented in more detail. The resistance mechanisms can be summarized in several unifying modes of action: increased activity of DNA repair mechanisms (on-target resistance); decreased cellular accumulation of the drug (through decreased uptake and increased export) and increased drug inactivation (pre-target resistance); defective pathways and the machinery that normally trigger apoptosis in response to DNA damage (post-target resistance); and activation of the survival pathways that compensate for the cisplatin-induced lethal signals (off-target resistance) [11,12,13,14]. More recently, the tumor microenvironment has been recognized as another relevant player in the acquisition of cisplatin resistance [15]. An important attribute of the mechanisms listed above is that they can be elicited by both genetic and epigenetic changes [16,17]. Epigenetic foundations of cisplatin resistance include DNA methylation that is changed globally and at numerous CpG sites in some tumor types (e.g., ovarian) on acquiring resistance [18,19]. Furthermore, cisplatin resistance is also linked to the action of noncoding RNAs [17]. However, the most interesting aspect of acquiring cisplatin resistance in regard to this review is the role of epigenetic readers, writers, and erasers involved in the post-translational histone modifications (such as acetylation and methylation) which affect global gene transcription. In line with this, lysine acetyltransferases, as well as the erasers of this mark, histone deacetylases, have been implicated in the development of cisplatin resistance, with these mechanisms discussed in more detail in previously published articles [16,17]. Enhancer of Zeste Homolog 2 (EZH2), the lysine methyltransferase that is the focus of this review, was also shown to influence resistance to cisplatin. Publications implying EZH2 in cisplatin resistance will be reviewed and presented in more detail in subsequent sections of this work. Considering the substantial amount of the literature that focuses on the effects of EZH2 activity in cisplatin resistance, modulation of EZH2 expression/activity could potentially be one solution that offers improvement in cisplatin therapy.

To conclude, combination therapy is expected to at least partially ameliorate two problems that are associated with cisplatin therapy: the toxic effects of cisplatin and the development of resistance. Numerous different approaches have been used to complement cisplatin action in anticancer treatments. Here, we focus on a combination that includes inhibition of EZH2, a catalytic subunit of the Polycomb Repressive Complex 2 (PRC2).

## 3. The Polycomb Repressive Complex 2 Related and Unrelated Roles of EZH2

Post-translational histone modifications play essential roles in establishing chromatin structure and subsequent gene expression. The Polycomb group of proteins have histone-modifying activities and are important epigenetic regulators. Among them, Polycomb Repressive Complex 2 catalyzes the methylation of the histone H3 lysine 27 (H3K27) to generate trimethyl-H3K27 (H3K27me3). This repressive epigenetic mark affects transcription of numerous target genes. The PRC2 contains either of the two existing Enhancers of Zeste Homolog 1 or 2 (EZH1/EZH2) orthologs, which is its catalytic subunit. Although partially overlapping in their function, EZH1 and EZH2 differ in a way that PRC2-EZH2 is abundant in highly proliferative cells and is mainly dedicated to the establishment of H3K27me3 mark, while PRC2-EZH1 is often present in non-dividing cells and restores this repressive mark [20,21]. In addition to EZH1/EZH2, the PRC2 contains Embryonic Ectoderm Development (EED) and Suppressor of Zeste 12 protein homolog (SUZ12) which, together with EZH2, comprise the core of the PRC2. The PRC2 also contains several cofactors such as Retinoblastoma (Rb)-Associated Protein 46/48 (RbAp46/48; sometimes considered to be a part of a core PRC2), AEBP2, and JARID2. The complexity of the PRC2’s interacting partners [22] and their dynamic exchange (“exchange phenomenon”) enables the precise control of gene activity, both spatially and in a controlled time-window [20]. It must be mentioned that EZH2 is also present in PRC3 and PRC4 complexes that methylate histones H3 and H1, respectively. However, its role in the PRC2 is the most prominent and the best studied.

EZH2 and the PRC2 machinery, in general, are evolutionarily conserved and identified in many species, from plants to flies and humans, sharing similar structural domains and motifs [22]. Besides being evolutionarily conserved, another important feature of the processes involved in the histone modifications is their reversibility. This means that, unlike genetic changes, these epigenetic marks are imposed and removed dynamically. That characteristic opens up avenues for their exploration and therapeutic modulations.

In addition to its master role in the PRC2, EZH2 displays a whole range of effects that are unrelated to the PRC2 and H3K27me3 [22,23,24]. For example, EZH2 has been shown to methylate STAT3 [25,26] and RORα [27]. Moreover, it can act as a transcription activator. That is, it enhances the activity of β-catenin by promoting its transition to the nucleus in mammary epithelial cells leading to hyperplasia [28]. Recently, EZH2 was reported as a part of a transcriptional complex consisting of EZH2, RNA-polymerase II, and nuclear actin. This complex plays a role in aberrant epithelial remodeling after injury [29]. In recent years, “non-canonical” roles of EZH2 have been relatively frequently reported, for example, in immune homeostasis [30], in promoting DNA repair [31], or in regulating the transcriptional effect of some other transcription factors [32], all adding to the complexity of downstream cellular processes that are triggered by this versatile protein.

## 4. EZH2 in Cancer

Since EZH2 impacts the expression of a variety of downstream target genes, it is not surprising that both the tumor promoting and tumor suppressive functions of EZH2 have been documented in the literature [33]. However, EZH2 and H3K27me3 have been linked to poor prognosis in many solid human cancers, emphasizing the important roles of EZH2/H3K27me3 in inhibiting the expression of different tumor suppressor genes which are the common EZH2 targets identified in cancer [22]. In this way, EZH2 is generally thought to remove the barriers against tumor growth and progression. However, this phenomenon is highly cell context dependent.

EZH2 itself is a frequent target of dysregulated expression in cancers. Aberrant EZH2 expression (usually upregulation) and activity in cancers is a consequence of genetic, transcriptional, post-transcriptional, and post-translational modifications which have been documented in various cancer types [22,34,35]. Generally, the increased activity of EZH2 potentiates cancer cell proliferation, survival, migration, invasion, and epithelial-to-mesenchymal transition (EMT). Additionally, EZH2 has been implicated in cancer stem cell biology [36,37]. It is thought that EZH2 maintains stem cell identity by globally repressing differentiation programs [36,38]. The fact that EZH2 activity is frequently upregulated in different cancer types imposes EZH2 as an appealing anticancer drug target [39,40]. Furthermore, its important roles in cancer pathogenesis (initiation and progression) and maintenance of cancer hallmarks, including metastasis, aberrant signaling and metabolism, drug resistance, and immunity regulation [22,39], suggest that EZH2 targeting would highly impact the cancer cell biology. Therefore, several strategies (outlined in the next section) have been developed to tackle the increased expression/activity of EZH2 in cancers.

It is expected that the inhibition or over-expression of EZH2 in each tumor type would influence different processes due to a multitude of cell-specific transcriptional targets. In line with that, overlapping, but also diverse, roles of EZH2 were described in prostate [24], breast [41,42], ovarian [43], lung [44,45,46], bladder [47], and many other cancer types. The generally accepted view is that EZH2 functions as an oncogenic factor in a majority of solid cancers, while it acts as a tumor suppressor in some blood malignancies (e.g., T cell acute lymphoblastic leukemia [48,49]).

The mechanisms by which EZH2 influences cancer cell biology are diverse and entangled. It is considered that EZH2 has a master regulatory function in controlling several potent signaling pathways in cancer. These include Wnt, Ras, NF-κB, BMP, Beta-adrenergic receptor, and Notch [38], which are affected primarily through the repression of the transcription of different signaling molecules that make important nodes in those pathways. By doing so, EZH2 is placed on the top of signaling machinery that controls cellular behavior. Therefore, its numerous roles in cancer cell biology were expected and have been confirmed by many scientific publications.

Another important highlight of EZH2 in cancers that needs to be emphasized is its decisive role in immune cells such as T cells, NK cells, dendritic cells, and macrophages which shape the tumor microenvironment [33,50,51,52]. EZH2 is suggested to be a main driver of cancer cells’ immunoediting which leads to immune escape. EZH2 is thought to downregulate immune recognition and activation and upregulate the immune checkpoints [53]. In this way, it creates an immunosuppressive tumor microenvironment and undermines the attempts of the immune system to eradicate cancer cells. Therefore, it was suggested that targeting EZH2 could potentially overcome the resistance to immunotherapy in several cancer types. A good example is lung cancer [54], which seems to be less susceptible to cancer immunotherapies (immunologically “cold” tumor) [53].

**Figure 1:**
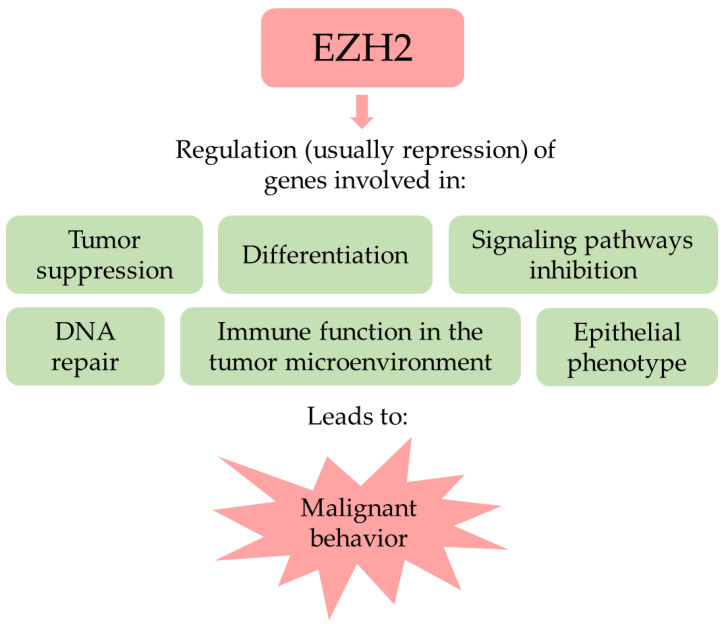
Generalized summary of EZH2 activities in cancer. Some of these roles offer an opportunity for therapeutic intervention, such as the modulation of the immune function. The roles of EZH2 in the epithelial–mesenchymal transition and DNA repair are complex and opposing and the cell context dependent.

In conclusion, as a master regulator of transcription, EZH2 influences many features of cancer (summarized in Figure 1). Additionally, its activity is upregulated in different cancer types. Taken together, these observations make EZH2 an attractive anticancer target.

## 5. EZH2 Inhibitors

Numerous studies have implicated EZH2 in cancer pathophysiology. Pharmacological inhibition of EZH2 has become a field for increased research efforts. Herein, the inhibitors that are used in combination therapy with cisplatin, in cancer in vitro and in vivo models (Table 1), are presented and described in more detail. Additionally, some general concepts on EZH2 inhibition are briefly outlined.

**DZNep** (3-deazaneplanocin A), the first-created EZH2 inhibitor, is a compound that was initially developed to target Ebola virus [55]. This molecule inhibits EZH2 action by acting as an S-adenosyl-L-homocysteine (SAH) hydrolase inhibitor. SAH is the byproduct generated after the transfer of methyl group from S-adenosyl-methionine (SAM), with the accumulation of SAH inhibiting SAM-mediated methyl transfer. In this way, DZNep represses SAM-dependent histone methyltransferase activity of EZH2 and globally inhibits histone methylation. However, DZNep did not demonstrate clinic utility due to its hydrophilic nature and rapid renal excretion [53].

In the past decade, several other potent and highly selective inhibitors of EZH2 have been created. They act as SAM-competitive inhibitors in the way that they partially occupy the site for the co-substrate SAM in the binding pocket of EZH2. Such molecules include **GSK126** (GSK2816126), which inhibits wild type and certain mutant EZH2 protein variants. This compound was initially developed for the treatment of lymphoma with EZH2-activating mutations [53]. However, there was a lack of activity of GSK2816126 in a phase I study in patients with advanced hematologic (mostly lymphomas) and solid tumors (NCT02082977), with no basis for its further exploration in a clinical setting [56].

**EPZ011989**, a potent, orally available EZH2 inhibitor with robust in vivo activity in a mouse xenograft model of human B cell lymphoma, was reported in 2015 [57]. Recently, this compound was shown to have a moderate in vivo efficacy in xenograft models of rhabdoid tumors [58]. 

Other SAM-competitive inhibitors of EZH2 include **GSK343**, **GSK926**, and **tazemetostat** (E7438/EPZ6438). Tazemetostat is the most prominent among them and is the most studied among the EZH2 inhibitors. It has also been tested in several clinical trials [39,53,59]. Moreover, in 2020, tazemetostat (Tazverik, Epizyme, Inc., Cambridge, MA, USA) was approved by the FDA for the treatment of epithelioid sarcoma and refractory follicular lymphoma [60]. The toxicity profile of tazemetostat is defined as manageable and well tolerated. The most common side effects include transient episodes of low energy, pain, decreased appetite, and mild gastrointestinal upset. It was estimated that less than 10% of patients experience adverse events that require discontinuation of therapy. The need for dose reductions is also rare [60]. However, an increase in secondary (new) cancers has been reported in patients (0.7%) who have been treated with tazemetostat. In general, other EZH2 inhibitors are well tolerated, but are also associated with relevant adverse events, including liver toxicity and fever. It is thought that these side effects are probably due to a lack of absolute tumor-related selectivity and consequential decrease of the basal expression of EZH2 in normal tissue [53].

Other strategies to inhibit EZH2 include dual EZH1/EZH2 inhibition (EZH1 complements EZH2 in mediating H3K27 methylation) and inhibitors that break the PRC2’s structure. Additionally, strategies that target EZH2 degradation have also been widely considered [39,40].

Experimental approaches such as RNA-based silencing (siRNAs and shRNAs) have also been widely used to modulate EZH2 signaling in cancer research. In the field of nanotechnology, there are attempts to deliver specific siRNAs together with drugs. There are several advantages of this approach in experimental cancer therapy that include circumventing some of the resistance mechanisms, enhancing toxicity for the tumor cells, and reducing adverse effects. Reports have suggested that such a therapeutic approach could gain success in combined platinum and EZH2-targeted therapy [61].

Investigatory efforts within a field of EZH2 inhibitors are continuing and it is expected that they will yield novel anticancer therapeutics.

## 6. Combined Effects of EZH2 Inhibition and Cisplatin in Anticancer Therapies

Both cisplatin and EZH2 inhibitors are explored in combination therapies. Combination therapies that include cisplatin have long been the focus of numerous research studies. More than 41,000 publications with the search term “cisplatin AND combination AND therapy” were retrieved from PubMed in August 2022 (time range 1975–2022). Among other combinations, cisplatin has been used with gemcitabine (a nucleoside analog) in bladder, pancreatic, cervical, ovarian, and non-small cell lung cancer, and malignant mesothelioma. The same combination is also used for some rare cancers, including locally advanced or metastatic biliary tract cancer [62]. Examples of other combined applications of cisplatin include those with paclitaxel (a cytoskeletal drug that targets tubulin), a combination that is used in a subset of patients with non-small cell lung cancer and ovarian cancer. For metastatic small cell lung cancer, cisplatin is used in combination with etoposide (topoisomerase inhibitor) as the standard first-line treatment [63]. Gemcitabine, paclitaxel, and etoposide are just a few examples of many combination therapies that include cisplatin that are either currently being studied or already approved for use. The data on combination therapies listed in this paragraph were retrieved from www.cancer.gov, accessed on 15 August 2022.

EZH2 inhibitors are also being explored in combination with other compounds. Examples of such compounds are docetaxel [64], etoposide [65], and temozolomide [66] (chemotherapy), PD-L1 and PD-1 inhibitors [67] (immunotherapy), antiandrogens [68] (endocrine therapy), and PARP [69] and HDAC inhibitors [70] (targeted therapy) [59,71]. These are expected to potentiate the beneficial effects of EZH2-targeted therapy.

The general mechanism and rationale for using EZH2 inhibitors together with cisplatin could potentially stem from the following possible scenario: as already explained, among other mechanisms of action, cisplatin damages DNA by generating DNA-platinum adducts which induce double-strand breaks (DSBs). These need to be repaired by the cell DNA repair machinery. However, this requires transient transcriptional repression of genes located in close proximity to DSBs [72]. Among other mechanisms involved, EZH2 contributes to the establishment of a transient repressive chromatin context that enables the DNA repair to proceed, which is the basis for survival of the cancer cell exposed to cisplatin. Therefore, it is expected that the blockade of EZH2 would contribute to the inability of the cell to repair its DSBs and increase the response rate to cisplatin.

In another scenario, EZH2 inhibitors (EZH2i) were shown to downregulate a set of DNA damage repair genes, especially those involved in the base excision repair (BER) pathway. In this way, EZH2i enhance the responses of prostate cancer cell lines to DNA-damaging agents and genotoxic stress. Activation of BER genes were shown to include FOXA1 action and its methylation by EZH2 and EZH2’s interaction with the transcriptional coactivator P300 [73].

These are only two possible general scenarios on how EZH2 could influence cisplatin resistance. Other suggested mechanisms are listed in the subsequent sections of this review.

The idea behind the combination approach to anticancer therapy is to use all agents simultaneously, or to intervene after the cancer cells acquired therapy resistance to one of the agents (sequential therapy). In the following sections and in Table 1, we present the literature that documented the roles of EZH2 inhibition either in targeting cisplatin-resistant cells and tumors, or giving the EZH2 inhibitors and cisplatin simultaneously. We also make a distinction between studies that showed positive and negative links between EZH2 and cisplatin resistance.

**Table 1:** Overview of preclinical studies of cisplatin combined with EZH2 inhibition.

Cancer Type	In Vitro Model	In Vivo Model	Cisplatin Concentration	EZH2 Silencing/ Inhibition	Possible Mechanism of Action	Effect of Combined Treatment	Ref.
Bladder cancer	Bladder cancer cell lines: HT1376, T24, and UM-UC-3	HT1376 cell line xenograft in nude mice	In vitro: 0.83 µM. In vivo: 3 mg/kg, intraperitoneally (i.p.), once per week	In vitro: EPZ011989 (1 µM). In vivo: EPZ011989 (500 mg/kg, oral gavage, every 12 h)	Inhibition of EZH2 induces natural killer cell-mediated differentiation and death in HT1376-derived xenografts.	Additive/synergistic: In vitro: Combined application of EPZ011989 and cisplatin caused G2/M arrest and reduced clonogenicity of T24 and UM-UC-3 cell lines. In vivo: Combined application reduced xenograft growth.	[74]
	Bladder cancer cell line: T24	/	2 μM	EZH2 siRNA	/	Additive/synergistic: EZH2 knockdown increased cisplatin cytotoxicity, while EZH2 overexpression reduced it.	[75]
Breast cancer	Breast cancer cell lines: MCF-7 and MDA-MB-231	MCF-7 xenograft in BALB/c nude mice	In vitro: 0.1–100 µM, mostly 10 µM. In vivo: i.p., 5 mg/kg, weekly	EZH2 siRNA	EZH2 knockdown increased expression of miR-381.	Additive/synergistic: In vitro: EZH2 knockdown sensitizes cells to cisplatin. In vivo: EZH2 knockdown sensitizes cells to cisplatin.	[76]
	Breast cancer cell lines: from BRCA1-deficient and BRCA1-proficient mice	BRCA1-deficient tumors in FvB/Ola mice	In vitro: 0.5 µM. In vivo: 3 mg/kg.	In vitro: GSK126 (8 µM). In vivo: GSK126 150 mg/kg daily	/	Additive/synergistic: In vitro: GSK126 increased cisplatin-induced growth inhibition only in BRCA1-deficient cells. In vivo: Combined application of GSK126 and cisplatin increased overall survival.	[77]
Cervical cancer	Cervical cancer cell line: HeLa	/	Range of concentrations (0–3333 µM)	EZH2 shRNA	/	Additive/synergistic: EZH2 knockdown reduced resistance to cisplatin.	[78]
Endometrial cancer	Endometrial cancer cell lines: Ishikawa, HEC1A, and KLE	/	Range of concentrations (0.1–100 µM)	EZH2 siRNA	EZH2 knockdown decreased the level of Peroxiredoxin 6 (PRDX6) protein.	Additive/synergistic: EZH2 knockdown sensitizes cell lines with higher EZH2 levels (Ishikawa and HEC1A) to cisplatin, but not cell line KLE which has lower levels of EZH2.	[79]
	Endometrial Cancer cell line: HEC1B	/	1 μM	GSK126 (7.5 µM)	/	Additive/synergistic: GSK126 increased cytotoxic effect of cisplatin.	[80]
Gastric cancer	Gastric cancer cell lines: MKN45 and MGC803	/	Range of concentrations (5–25 µM)	EZH2 siRNA	EZH2 inhibition reduced the activity of PI3K/AKT pathway.	Additive/synergistic: EZH2 knockdown enhances cisplatin-induced apoptosis.	[81]
Head and neck cancer	Head and neck squamous cell carcinoma cell lines: FaDu andSNU1041	/	Range of concentrations (5–50 µM)	EZH2 siRNA	EZH2 knockdown reduced N-cadherin and vimentin and increased E-cadherin expression.	Additive/synergistic: EZH2 knockdown increased cytotoxic effect of cisplatin.	[82]
	Head and neck cancer cell line: SCC-11	/	33.3 µM	EZH2 siRNA	/	Additive/synergistic: EZH2 knockdown increased cancer cells’ sensitivity to cisplatin.	[83]
	Head and neck cancer cell lines: CNE and 8F cells	/	Range of concentrations (0–64 µM)	GSK126 (1 µM)	EZH2 suppresses the nucleotide excision repair by silencing XPA.	Antagonistic: GSK126 doubled the resistance to cisplatin.	[84]
Liver cancer	Liver cancer cell lines: HepG2 and SNU449	/	Range of concentrations (0–20 µM)	EZH2 siRNA	miR138 targets the EZH2/EMT axis.	Additive/synergistic: EZH2 knockdown enhanced sensitivity to cisplatin.	[85]
Lung cancer	Lung cancer cell line: A549	/	Range of concentrations (2–16 µM), mostly 4 µM.	EZH2 siRNA, Tazemetostat (40 µM)	Knockdown of EZH2 increased levels of cleaved caspase 3 and 9, E-cadherin and reduced expression of N-cadherin and vimentin.	Additive/synergistic: EZH2 knockdown or inhibition with tazemetostat increased cisplatin cytotoxicity.	[86]
		/	Range of concentrations (20–140 µM)	shRNA	EZH2 knockdown reduced MRP1 mRNA levels.	Additive/synergistic: EZH2 knockdown increased cancer cells’ sensitivity to cisplatin.	[87]
	Lung cancer cell lines: H128 and H146	H128 cells xenograft in Nu/Nu mice	In vitro: Range of concentrations (5–25 µM). In vivo: 2.5 mg/kg; 2 times weekly, for 4 weeks.	In vitro: EZH2 siRNA, DZNep (2.5–5 µM), Tazemetostat (1 µM). In vivo: DZNep (2.5 mg/kg; 2 times weekly)	EZH2 interacts with and stabilizes components of nucleotide excision repair (DDB2).	Additive/synergistic: In vitro: siRNA and DZNep caused sensitization of cancer cells to cisplatin. No significant effect caused by combining cisplatin and tazemetostat. In vivo: Reduced tumor growth in mice treated with DZNep and cisplatin compared to individual agents.	[31]
	Lung cancer cell lines: H1299, H23, and H460	/	1–2 µM	EZH2 shRNA	EZH2 silencing upregulates PUMA, a proapoptotic protein.	Additive/synergistic: EZH2 knockdown increased cytotoxic effect of cisplatin.	[88]
	Lung cancer cell lines: A549, HCC4006, and H2073	/	Range of concentrations (0–80 µM).	DZNep (2.5 µM) EZH2 siRNA	/	Additive/synergistic: DZNep and EZH2 knockdown sensitized cells to cisplatin.	[89]
	Lung cancer cell line: H1299	/	66.6 µM	DZNep (range 0–10 μM)	/	Additive/synergistic: DZNep increased cisplatin cytotoxicity in H1299 cell line.	[90]
Osteosarcoma	Osteosarcoma cell lines: HOS and 143B	/	Range of concentrations (0.2–1.6 µM), but mostly 1 µM	Tazemetostat (10 µM)	Reductions in H3K27me3 levels induce PRKCA and MCL1 expression, BCL2 phosphorylation and activation of RAF/ERK/MAPK cascades.	Antagonistic: Tazemetostat increased resistance to cisplatin, leading to lower rates of apoptosis.	[91]
Ovarian cancer	Ovarian cancer cell line: HeyA8	/	1.25 µM	EZH2 siRNA	Reduced β-catenin levels after EZH2 silencing.	Additive/synergistic: EZH2 knockdown decreased proliferative capacity of cells exposed to cisplatin.	[92]
	Ovarian cancer cell lines: IGROV1, PEO1, and PEO4	/	Range of concentrations (0–80 µM)	miR-137, GSK343 (0–25 µM), EZH2 siRNA	miR-137 mediates the functional link between c-Myc and EZH2 that regulates cisplatin resistance.	Additive/synergistic: siRNA-EZH2 and GSK343 sensitized resistant cell lines to cisplatin. EZH2-depleted cells treated with cisplatin showed an increase in cell apoptosis (elevated level of cleaved PARP).	[93]
	Ovarian cancer cell line: SKOV3	/	25.53 µM	EZH2 shRNA	EZH2 silencing upregulated p14, p16, p53, and pRB.	Additive/synergistic: EZH2 knockdown increased cytotoxic effect of cisplatin.	[94]
	Ovarian cancer cell lines: A2780 and ES2	/	2, 4, or 8 µM	EZH2 shRNA	Reduced EZH2 levels increased cisplatin intake. High EZH2 levels promoted degradation of CTR1.	Additive/synergistic: shRNA sensitized cells to cisplatin. Overexpression of EZH2 leads to increased resistance to cisplatin.	[95]
	Ovarian cancer cell line: A2780	A2780 xenograft in female BALB/c nude mice	In vitro: range of concentrations, mostly 10 µM. In vivo: 2 mg/kg tail vein injection	EZH2 siRNA (bound to Fe3O4 particles with cisplatin prodrug)	/	Additive/synergistic: In vitro: EZH2 knock-down increased sensitivity to cisplatin. In vivo: siRNA-EZH2 coated nanoparticles and cisplatin inhibited tumor growth more than nanoparticles with control siRNA and cisplatin.	[61]
			In vitro: Range of concentrations (10–120 µM). In vivo: 6 mg/kg, i.p., once, on day 0	EZH2 shRNA	/	Additive/synergistic: In vitro: EZH2 knock-down increased sensitivity to cisplatin. In vivo: EZH2 knockdown combined with cisplatin led to greater reduction of tumor growth than cisplatin alone.	[96]
		/	Range of concentrations (2–165 µM)	EZH2 shRNA	Depletion of EZH2 in cisplatin-resistant cells reduced BRCA1 expression.	Additive/synergistic: EZH2 knockdown increased cancer cells’ sensitivity to cisplatin.	[97]
Testicular cancer	Testicular cancer cell lines: NT2/D1, 2102EP, and 833K	/	Range of concentrations (0.1–10 µM)	GSK126 (1µM)	/	Antagonistic: Inhibition of EZH2 with GSK126 increased cancer cell resistance to cisplatin.	[98]

### 6.1. EZH2 as a Potentiator of Cisplatin Resistance

#### 6.1.1. Ovarian Cancer

In recent decades, cisplatin has been among the most prominent drugs for the treatment of ovarian cancer and the prognosis for women with ovarian cancer can be defined by their tumor’s response to cisplatin [99,100]. The first publication studying the role of EZH2 in cisplatin resistance was a report by Hu et al. [96]. They documented that EZH2 was overexpressed in cisplatin-resistant ovarian cancer cells (A2780/DDP) compared with cisplatin-sensitive cells (A2780). Moreover, they showed that loss of EZH2 in vitro and in vivo (A2780/DDP cells in BALB/c nude mice) enhanced sensitivity to cisplatin. The authors suggested that the effect was mediated through a “canonical” EZH2 role, that is, H3K27 methylation.

Further studies focused in more detail on the potential mechanism and targets of EZH2 in cisplatin-resistant ovarian cancer cells. They suggested that repression of endoribonuclease Dicer, which is an EZH2 target in the cisplatin-resistant A2780 ovarian cancer cell line, could be among the potential mediators of cisplatin resistance [101]. Another study [97] implicated the role of BRCA1, downstream of EZH2, in cisplatin-resistant sub-line A2780/DDP. Mutations of BRCA1 play an important role in the development of ovarian cancer [102]. In the cited study [97], the depletion of EZH2 in parental A2780 cells upregulated BRCA1 protein expression and its nuclear translocation, albeit unexpectedly, decreased BRCA1 mRNA level. However, depletion of EZH2 in A2780/DDP cells reduced BRCA1 expression at both mRNA and protein levels. Moreover, downregulation of EZH2 or BRCA1 sensitized A2780/DDP cells to cisplatin, which contradicts BRCA1’s tumor-suppressing role but is in line with the concept that its upregulation is associated with DNA repair-mediated resistance to cisplatin [103]. An additional explanation for BRCA1’s downregulation-mediated enhancement of cisplatin sensitivity comes from its recently discovered role in the regulation of ovarian cancer cells’ metabolism. It has been demonstrated that BRCA1 deficiency drives metabolic reprogramming by upregulating nicotinamide N-methyltransferase (NNMT), sensitizing ovarian cancer cells to agents that inhibit energy metabolism [104]. As cisplatin negatively affects both the mitochondrial tricarboxylic acid (TCA) cycle and glycolysis [105], it is conceivable that BRCA1’s deficiency-mediated metabolic reprogramming would sensitize cells to this chemotherapeutic. 

Further delineation of EZH2’s role in the cisplatin resistance of ovarian cancer has shown that EZH2 inhibition does not induce apoptosis, but it can suppress the cisplatin-resistant human ovarian cancer cell line SKOV3/DDP autophagy and reverse resistance to cisplatin. In addition, the authors have shown that EZH2 inhibition increased the expression of the cellular senescence signaling proteins p14ARF, p16INK4a, p53, pRb, and p21, and decreased the expression of CDK1, CDK2, and the levels of H3K27me3 [94]. In bioinformatic studies that compared the transcriptomes of cisplatin-sensitive and -resistant A2780 cells, EZH2 mRNA was found to be downregulated in cisplatin-sensitive cells. Additionally, 34 of its interacting genes showed differential expression, indicating the potential broad involvement of EZH2 action in cisplatin resistance [106,107].

To the best of our knowledge and belief, no reports exist on the combined application of EZH2 inhibitors and cisplatin in humans with ovarian cancer. However, the study by Sun et al. analyzed EZH2 protein expression levels in 84 tumor tissue specimens of ovarian cancer patients. They showed that EZH2 staining (the tissue microarray and immunohistochemical assay) in the cisplatin-chemoresistant group of patients was much stronger than in the chemosensitive group, highlighting the fact that patients with high EZH2 levels tend to have poor responses to cisplatin. In a cell culture model (A2780 and ES2 cells), the authors further showed that EZH2 depletion seems to increase cellular platinum uptake contributing to the reversal of resistance. More precisely, the downregulation of EZH2 protected CTR1 (Copper Transporter 1), which is closely related to cisplatin resistance, from cisplatin-induced proteasomal degradation [95].

In addition to studies that focus on the events downstream of EZH2, several studies describe the roles of microRNAs and lncRNAs in a cascade that leads to changed EZH2 expression or activity and a subsequent modulation of cisplatin resistance in ovarian cancer. The roles of miR-506-3p [92], lncHOTAIR [108,109], miR-138-5p [109], miR-137 [93], H19 lncRNA [110], miR-101 [111], and let-7e [112] have been implicated.

#### 6.1.2. Lung Cancer

Non-small cell lung cancer (NSCLC) is the most common type of lung cancer for which the application of first-line platinum-based chemotherapy is used, among other treatment options. On a cohort of 360 patients with advanced NSCLC, Xu et al. showed that the expression of EZH2 in biopsy samples predicts both chemoresistance to cisplatin-based chemotherapy (EZH2-negative patients respond better to chemotherapy) and survival (positive EZH2 expression was correlated with poor survival) [113]. In line with this, Liu et al. confirmed that high levels of EZH2 protein were associated with a worse overall survival rate in a group of 49 patients with adenocarcinoma and 60 patients with squamous cell carcinoma. Moreover, they showed that in NCI-H1299, NCI-H23, and NCI-H460 lung cancer cell lines, EZH2 silencing sensitized cells to cisplatin-induced apoptosis. The authors suggested that the possible mechanism could be mediated through the induction of the expression of the pro-apoptotic protein PUMA (P53 Upregulated Modulator of Apoptosis), since EZH2 binds to the PUMA promoter and represses its expression [88]. In a recent work on A549 cell model, the authors showed that EZH2 knockdown enhanced the sensitivity of cancer cells to cisplatin by inhibiting viability and promoting apoptosis [86]. Two other reports supported these conclusions [87,114].

Another subset of lung cancer, small cell lung cancer (SCLC), has initially shown a good response to cisplatin and etoposide (topoisomerase II inhibitor) which are the standard first-line treatments for metastatic SCLC. However, in a majority of cases, tumors become rapidly chemoresistant, leading to death within one year. In their recent study, using patient-derived tumor xenografts, Gardner et al. showed that chemoresistance to cisplatin and etoposide in SCLC is mediated by the suppression of SLFN11 (Schlafen Family Member 11), a protein that inhibits DNA replication and promotes cell death in response to DNA damage. The authors showed that EZH2, which is induced by cytotoxic chemotherapy, deposits repressive chromatin marks in the SLFN11 gene body, decreases SLFN11 expression, and promotes chemoresistance [63]. Further delineation of EZH2 mechanisms in SCLC cisplatin resistance, by Koyen et al., revealed the non-catalytic and PRC2-independent role of EZH2 in stabilizing DDB2 (Damage Specific DNA Binding Protein 2). EZH2 promotes DDB2 stability by impairing its ubiquitination, which promotes nucleotide excision repair and directs cisplatin resistance in NCI-H128 cells. However, NCI-H128 cells are sensitized to cisplatin through EZH2 depletion but not through SAM competitive inhibition. In the same study, the authors showed that targeting EZH2 with DZNep inhibitor strongly sensitized SCLC cells and tumors (Nu/Nu mice with NCI-H128 lung cancer xenografts) to cisplatin [31]. In another study that used publicly available datasets and bioinformatic tools, EZH2, along with five other genes, was detected as a potential prognostic and predictive biomarker for response to immune-checkpoint inhibitors treatment in patients with SCLC. In this study, low expression of MCM2, EZH2, CENPK, and CHEK1 was correlated with increased sensitivity to cisplatin [115].

The upstream signaling events that lead to EZH2 activation and subsequent cisplatin resistance in lung cancer (mainly NSCLC) possibly include the SETD1A/Wnt/β-catenin feedback loop [116], VEGF/VEGFR2 signaling [89], and AFAP1-AS1 [117]. Further, a recent study using cells, xenografted mice, patient-derived xenografts, and needle biopsies from 15 lung adenocarcinoma patients has suggested that cisplatin-HOXB13-ABCG1/EZH2/Slug axis could be a novel mechanism underlying cisplatin resistance and metastasis after chemotherapy. Among other proteins, HOXB13 upregulated ABCG1, EZH2, and SLUG by directly binding to their promoters [118]. Of special interest is the study on linc00665, which was shown to interact with EZH2 and recruit it to the promoter region of CDKN1C, reducing CDKN1C expression and affecting cisplatin sensitivity [119]. This study showed that not only is EZH2 expression/activity crucial, but so is the availability of its binding partners, which lead EZH2 to the targeted spots of DNA.

In Section 4, we discussed the immunosuppressive role of EZH2 and provide lung cancer as an example. The programmed cell death protein 1 (PD-1)/programmed cell death protein ligand 1 (PD-L1) axis leads to the exhaustion of T-cell immunity in chronic infections and tumors. That axis is the immune modulator that has been extensively studied in anticancer treatments, including NSCLC [120]. Recently, in a study that included NSCLC patients and in vivo models, it was suggested that cisplatin treatment could synergize with PD-1/PD-L1 blockade to increase the clinical response in NSCLC [121]. A combination of cisplatin and anti-PD-1 antibody also enhanced the treatment efficacy in some other cancer types, including advanced esophageal squamous cell carcinoma [122]. These results suggest that both cisplatin and EZH2i could potentially act in a concerted way to stimulate an immune response in NSCLC and other cancer types.

#### 6.1.3. Breast Cancer

BRCA1 (Breast Cancer Type 1 Susceptibility Protein) gene mutations in the germline are a hallmark of hereditary breast and ovarian cancers. Among other roles, this protein is involved in maintaining genomic stability through its role in DNA damage repair. In breast cancer, cisplatin is of special use for treating tumors that are deficient in BRCA1, since they are hypersensitive to DNA double-strand break (DSB)-inducing compounds. In a recent study, using IHC or RNA sequencing on a cohort of 497 breast cancers, Puppe et al. showed that EZH2 is expressed at significantly higher levels in BRCA1-deficient breast cancers, classified through BRCA1 mutation status, BRCA1-promoter hypermethylation, or a BRCA1-like DNA copy number profile. Moreover, EZH2 overexpression identifies patients with breast cancer who benefit from a high-dose DSB-inducing platinum-based chemotherapy. In an accompanying in vivo experiment, EZH2 inhibition was shown to improve the antitumor effect of platinum drugs in BRCA1-deficient murine mammary tumors [77]. In line with this, a further study showed that EZH2 was upregulated in cisplatin-resistant breast cancer tissue (tumor tissue from 48 breast cancer patients) and cell lines (MCF-7/CDDP and MDA-MB231/CDDP compared with parental cells). Analysis of the expression of EZH2 in breast cancer tissue from the TCGA database and in the mentioned authors’ own cohort (RT-qPCR) showed that breast cancer patients with high EZH2 expression had a poor prognosis. Moreover, EZH2 silencing enhanced the sensitivity to cisplatin of MCF-7/CDDP (in vitro and in vivo) and MDA-MB-231/CDDP cisplatin-resistant cell lines. The authors also showed that EZH2 contributes to cisplatin resistance through epigenetically silencing miR-381 [76]. Further delineation of this mechanism documented the involvement of lncSNHG1, which was found to interact with EZH2 and enhance its inhibition of miR-381 transcription in MCF-7 and MDA-MB-231 cells [123]. In another study on the role of EZH2 in breast cancer cisplatin resistance, it was shown that Salvianolic acid B promoted vascular normalization in mouse models of breast cancer, which subsequently improved drug delivery and response to cisplatin. The mechanism behind this effect is suppression of EZH2-driven cytokines which disrupt the endothelial junctions with diminished expression of VE-cadherin [124].

From the events that lead to EZH2 activity in cisplatin resistance, it was recently shown that targeting MUC1-C, a primarily transmembrane oncoprotein which may be localized in the nucleus, sensitizes triple-negative breast cancer (TNBC) cells to cisplatin treatment. In addition to other roles in this context, MUC1-C regulates nuclear localization of EZH2 and its function in the formation of H3K27me3, which is necessary for the repair of double-strand breaks [125]. Other studies that converge to the EZH2 role in breast cancer cisplatin resistance include the overexpression of SERPINA3 (serpin peptidase inhibitor, clade A, member 3), which reduces the sensitivity of TNBC cells to cisplatin and upregulates EZH2 [126].

#### 6.1.4. Muscle-Invasive Bladder Cancer

Muscle-invasive bladder cancer is a cancer subtype that spreads into the detrusor muscle of the bladder. The treatment options include radical cystectomy and neoadjuvant chemotherapy with the gemcitabine–cisplatin combination. The first report dealing with cisplatin resistance in muscle-invasive bladder cancer used the collagen gel droplet-embedded culture-drug sensitivity test. The authors showed that EZH2 knockdown increased the sensitivity of the T24 bladder cancer cells to gemcitabine and cisplatin, while EZH2 overexpression decreased their sensitivity [75]. In a further study, Ramakrishnan et al. showed that muscle-invasive bladder cancer cells’ HT1376 with KMD6A (histone demethylase aimed against H3K27me3) and SWI/SNF family member mutations are sensitive to EZH2 inhibition alone and in combination with cisplatin (both in an in vitro and in an in vivo HT1376 xenograft model). The sensitivity, the authors showed, is mediated through increased natural killer (NK) cell-related signaling, which potentiates tumor cell differentiation and cell death [74]. For the events upstream of EZH2 activation in bladder cancer models, it was shown that lncRNA TUG1 promoted cisplatin resistance via regulation of the CCND2 through EZH2-associated silencing of miR-194-5p [127].

#### 6.1.5. Head and Neck Squamous Cell Carcinoma

Head and neck squamous cell carcinoma (HNSCC) develop from the mucosal epithelium in the oral cavity, nasopharynx, oropharynx, hypopharynx, and larynx. It accounts for more than 90% of the cancers of the head and neck and is among the most common cancers by incidence worldwide [128,129]. The standard chemotherapy regimens for stage III or IV HNSCC patients include cisplatin, 5-fluorouracil (5-FU), and docetaxel/paclitaxel [130]. However, as is the case with many other cancer types, chemotherapy resistance is the barrier to their use in HNSCC patients.

In a retrospective analysis performed on 90 HNSCC patients, Chang et al. showed that high EZH2 expression was correlated with poor survival outcome. Moreover, EZH2 suppression increased cisplatin cytotoxicity in HNSCC FaDu and SNU1041 cells [82]. In another study based on human HNSCC samples, cell lines, and xenograft tumors (orthotopic model of UM1 cells in BALB/c nude mice), the authors suggested that STAT3/HOTAIR/EZH2 axis might be a potential therapeutic target for combination therapy of cisplatin and cetuximab in HNSCC patients with PI3K activation [131]. By studying the effects downstream of cisplatin-induced cell death and induction of the phospho-ΔNp63α (a key regulator of the cisplatin-induced microRNAome in cancer cells [132]), Huang et al. [83] showed that cisplatin induced the expression of EZH2. Moreover, siRNAs against EZH2 rendered the resistant SCC-11M cells to be more sensitive to cisplatin-induced cell death. In this model, EZH2 is part of a network of several transcription coactivators and corepressors that are activated in HNSCC cells exposed to cisplatin.

#### 6.1.6. Gastric Cancer

Platinum drugs (cisplatin and oxaliplatin) are the first-line treatment in chemotherapy for gastric cancer [133]. However, drug resistance is a major obstacle in gastric cancer chemotherapy. Silencing EZH2 through siRNA reversed cisplatin resistance in cisplatin-resistant AGS/DDP gastric cancer cells [114]. While the downstream mechanisms of this action still need to be elucidated, the mechanisms that converge on EZH2 in gastric cancer cisplatin resistance have been more explored. These include the action of PCAT-1 (Prostate Cancer-Associated Transcript 1), which epigenetically silenced PTEN (Phosphatase and Tensin Homolog) via recruiting EZH2 in BGC823/CDDP and SGC7901/CDDP cisplatin-resistant gastric cancer cell lines [134]. Another study implicated the role of lncRNA HOXD cluster antisense RNA 1 (HOXD-AS1), whose knockdown in cisplatin-resistant gastric cancer cells (BGC823/DDP and SGC7901/DDP) increased their sensitivity to cisplatin treatment. Mechanistically, HOXD-AS1 contributed to the epigenetically silencing of PDCD4 through binding to EZH2 [135]. In a recent study, the authors showed that lncRNA UCA1 promoted the cisplatin resistance of MKN45 and MGC803 gastric cancer cells by recruiting EZH2, activating the PI3K/AKT pathway, and inhibiting cisplatin-induced apoptosis [81]. 

#### 6.1.7. Endometrial Cancer

Cisplatin and doxorubicin are conventional drugs for the treatment of endometrial cancer. In 11 endometrial cancer cell lines and 52 clinical endometrial cancer specimens, EZH2 mRNA (RT-qPCR) was shown to be significantly overexpressed in cancer cells and tissue compared with the corresponding normal control cell lines and tissue. RNA-sequencing data from the TCGA database indicated that patients with high EZH2 expression have significantly poorer progression-free survival (this was in agreement with the immunohistochemical analysis of the tissue microarray specimens) and overall survival (OS). Additionally, EZH2 inhibitor GSK126 showed additive effects with cisplatin or doxorubicin in HEC1B cells [80]. These findings were confirmed in a further study that showed that increased EZH2 expression was significantly correlated with decreased disease-free and OS of patients based on immunohistochemical staining of 150 samples of endometrial cancer tissue. Moreover, EZH2 silencing enhanced the cytotoxicity of cisplatin and taxanes in HEC1A and Ishikawa endometrial cancer cell lines [79].

#### 6.1.8. Other Cancer Types

Cervical cancer is among the most common female malignancies and chemotherapeutic drug resistance is a major problem in cervical cancer therapy. Cai et al. showed that inhibiting the endogenous EZH2 reversed cisplatin resistance and increased the cisplatin sensitivity in cisplatin-resistant HeLa/DDP cells. The authors suggested that the effect is mediated partly by the upregulation of Dicer expression [78].

From the mechanisms that converge on EZH2 in cisplatin resistance, upregulation of miR-138 was shown to enhance sensitivity to cisplatin in hepatocellular carcinoma via regulation of EZH2 [85]. In esophageal squamous cell carcinoma, it was shown that TUG1 (long non-coding RNA Taurine Upregulated Gene 1) mediated cisplatin resistance by epigenetically suppressing PDCD4 (Programmed Cell Death 4) expression through recruiting EZH2 [136]. 

### 6.2. EZH2 as a Negative Regulator of Cisplatin Resistance

Although a substantial amount of the literature suggests the beneficial effects of EZH2 inhibition in combination with cisplatin therapy in different cancer types, reports exist documenting the inverse relationship of EZH2 and cisplatin resistance. 

Testicular germ cell tumors (TGCT) are sensitive to cisplatin, which contributes to their good prognosis. However, a subset of patients develop resistance to platinum-based therapies [137]. In a recent report, Singh et al. showed that acquired cisplatin resistance in NT2/D1, 833K, and 2102EP testicular cancer cell lines was associated with elevated expression of genes that are usually repressed by H3K27 methylation. Inhibition of EZH2 in those cell lines led to cisplatin resistance, while inhibition of H3K27 demethylase sensitized cells to cisplatin. Furthermore, studies on cisplatin-resistant cells, compared with parental controls, showed that cisplatin resistance was linked to decreased H3K27 methylation, H2A-K119 ubiquitination, and decreased expression of BMI1 and EZH2. Finally, a H3K27me3-based gene signature predicted cisplatin sensitivity in TGCT cells (with the exception of the cell line NT2/D1-B3) and disease-free survival in TGCT patients (based on RNA-sequencing and clinical data from TCGA) [98]. These results clearly demonstrate that EZH2 and the PRC2 are negative regulators of cisplatin resistance in TGCTs.

In another study, cisplatin-resistant osteosarcoma tissue showed lower H3K27me3 levels than sensitive tissue in immunohistochemical examination, including four cisplatin-sensitive and eight cisplatin-resistant specimens. Moreover, increased levels of H3K27me3 were shown to sensitize osteosarcoma cells and tumors to cisplatin. Namely, inhibition of the EZH2 in 143B and HOS osteosarcoma cells decreased H3K27me3 levels and led to cisplatin resistance. Furthermore, the inhibition of the demethylases KDM6A and KDM6B increased H3K27me3 levels in osteosarcoma cells and reversed cisplatin resistance in vitro and in vivo (143B cells in athymic nude mice). The studies on the involved mechanisms revealed the roles of PRKCA and MCL1, inactivation of RAF/ERK/MAPK cascades, and decreased phosphorylation of BCL2 [91]. However, contrary to this, in an earlier study, it was shown that miR-138 targets EZH2 and elevates cisplatin-induced apoptosis in MG-63 and U2OS osteosarcoma cells [138]. These two studies indicate that caution is needed when deducing general conclusions on the roles of EZH2 in the cisplatin resistance of each tumor type.

The examples of opposing roles of EZH2 are also documented in non-small cell lung and head and neck cancer cells. Contrary to what was elaborated earlier on the role of EZH2 in cisplatin resistance, in a recent study, the authors showed that long non-coding RNA ACTA2-AS1 inhibited the cisplatin resistance of NSCLC cell lines through suppressing TSC2 expression by recruiting EZH2 to the TSC2 gene promoter [139]. Further, in nasopharyngeal carcinoma CNE and 8F cell lines, it was shown that EZH2 suppresses the nucleotide excision repair via silencing of the XPA gene (DNA damage recognition and repair factor) through H3K27 trimethylation. Consequently, cells with inhibited EZH2 showed increased resistance to DNA damaging agent cisplatin [84].

**Figure 2:**
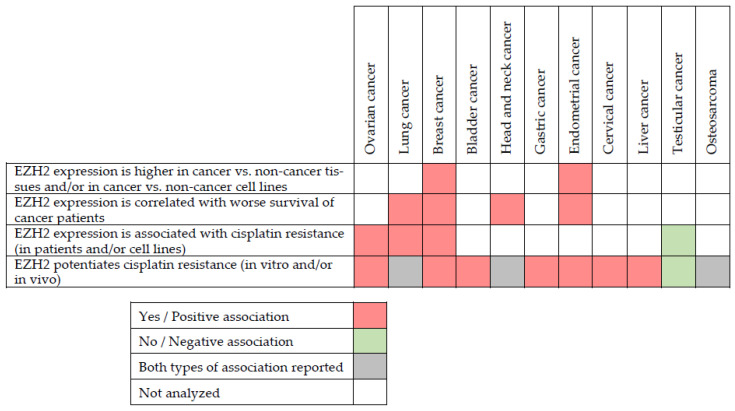
Summary of studies on EZH2 and cisplatin resistance in different cancer types.

With such a multitude of targets, it is hard to untangle each of the multiple roles of EZH2 in the cisplatin resistance of each cancer type. However, certain roles are expected to be superior with more impact on downstream processes and with the power to override the roles of some other players involved. Moreover, the cell context should always be considered when drawing conclusions on EZH2 action. The effects of EZH2 on cisplatin resistance in different cancer types are summarized in Figure 2.

## 7. EZH2 Inhibition and Cisplatin Toxicity

In addition to circumventing resistance, as previously mentioned, one of the benefits of combination therapy is the amelioration of toxic effects. The role of EZH2 inhibition in alleviating cisplatin toxic effects on the kidneys is the subject of several publications. However, while Ni et al. suggest that DZNep, via restoration of E-cadherin expression, protects against cisplatin-induced renal tubular cell apoptosis (mouse renal proximal tubular epithelial cell, mTEC) and acute kidney injury (male C57BL/6J mice) [90], Chen et al. found that DZNep promoted apoptosis of renal tubular epithelial cells, both in the presence and absence of cisplatin. In their study on NRK-52E cells, cisplatin induced the activation of mTORC1 and mTORC2 and apoptosis, while DZNep inhibited mTORC1 and mTORC2 activity and potentiated cisplatin-induced cell apoptosis [140]. In another study, Wen et al. found that cisplatin-treated mice displayed serious acute kidney injury symptoms (kidney dysfunction and kidney histological injury) which were co-occurring with EZH2 upregulation in the nuclei of renal tubular epithelial cells. The application of a selective EZH2 inhibitor dose-dependently alleviated these symptoms and demonstrated an anti-inflammatory effect in the kidneys of cisplatin-treated mice, via upregulating RKIP (Raf Kinase Inhibitor Protein) and blocking NF-κB p65 signaling [141]. Finally, in a very recent publication, Yu et al. found that the inhibition of the PRC2 via targeting EED protected against cisplatin-induced acute kidney injury. Applying a highly selective PRC2 inhibitor improved renal function, attenuated cisplatin-induced pathological changes, and reduced apoptosis of renal tubular cells in a murine model (male C57BL/6J mice) of cisplatin-induced acute kidney injury [142].

## 8. Combined Effects of EZH2 Inhibition and Other Platinum Compounds in Anticancer Therapies

Other common platinum-based drugs include oxaliplatin and carboplatin, with both widely used for cancer treatment. Among other cancer types, oxaliplatin is also used to treat colorectal cancer. Several upstream molecules were shown to converge on EZH2 in colorectal cancer, potentiating its resistance to oxaliplatin. Such molecules include MALAT1 [143], TRIM25 [144], a KLF4/PiHL/EZH2/HMGA2 regulatory axis [145], and downregulation of MEIS1 mediated by ELFN1-AS1/EZH2/DNMT3a axis [146]. However, a recent publication suggests that elevated H3K27me3 levels sensitize colorectal cancer to oxaliplatin. In their cited work [147], the authors showed that the reduced levels of H3K27me3 achieved by EPZ-6438 EZH2 inhibitor promoted HCT116 and SW620 colorectal cancer cells’ resistance to oxaliplatin.

Standard treatment for serous ovarian cancers includes carboplatin and paclitaxel. A recent report using 17 primary serous ovarian cancer patients and in vitro models showed that EZH2 loss drives resistance to carboplatin and paclitaxel in serous ovarian cancers that express Ataxia Telangiectasia Mutated (ATM) [148].

## 9. Conclusions

Although the role of EZH2 is context dependent, a substantial amount of the literature on preclinical models suggests that targeting EZH2 in combination with cisplatin chemotherapy could have beneficial effects in the treatment of lung, ovarian, and breast cancers. In addition to circumventing cisplatin resistance, this combination could potentially alleviate cisplatin toxic effects on the kidneys. Although there are encouraging results in HNSCC, bladder, gastric, cervical, and endometrial tumors, more studies, especially those conducted in a clinical setting, are needed to confirm the beneficial effects of a combined EZH2-targeting and cisplatin therapy in these and other tumor types and to shed light on potential mechanisms of action. However, in TGCT tumors, such a combination would not be recommended since EZH2 seems to have an opposing role to those described in a majority of other tumors, that is, its downregulation is positively associated with cisplatin resistance.

With respect to possible mechanisms which mediate EZH2 inhibition in cisplatin-resistant tumors, it is clear from this overview that they are diverse and include many upstream regulators (including miRNAs and lncRNAs) as well as downstream executors. Very often, EZH2 is associated with the aberrant expression of proteins with a major role in a given cancer, as, for example, BRCA1 in ovarian and breast cancers. However, a variety of other proteins have been documented to mediate the role of EZH2 in cisplatin resistance. Given the versatility of the EZH2 protein and its numerous targets, such a broad activity is to be expected.
